# Pituitary function within the first year after traumatic brain injury or subarachnoid haemorrhage

**DOI:** 10.1007/s40618-016-0546-1

**Published:** 2016-09-26

**Authors:** A. Tölli, J. Borg, B.-M. Bellander, F. Johansson, C. Höybye

**Affiliations:** 1 Department of Clinical Sciences, Danderyd Hospital, Karolinska Institutet, 182 88 Stockholm, Sweden; 2 Department of Clinical Neuroscience, Section for Neurosurgery, Karolinska Institutet, Stockholm, Sweden; 3 Medical Library, Danderyd University Hospital, Stockholm, Sweden; 4 Department of Molecular Medicine and Surgery, Karolinska Institutet, Stockholm, Sweden; 5 Department of Endocrinology, Metabolism and Diabetology, Karolinska University Hospital, Stockholm, Sweden

**Keywords:** Pituitary, Hormone disturbance, Traumatic brain injury, Subarachnoidal haemorrhage

## Abstract

**Purpose:**

Reports on long-term variations in pituitary function after traumatic brain injury (TBI) and subarachnoid haemorrhage (SAH) diverge. The aim of the current study was to evaluate the prevalence and changes in pituitary function during the first year after moderate and severe TBI and SAH and to explore the relation between pituitary function and injury variables.

**Methods:**

Adults with moderate and severe TBI or SAH were evaluated at 10 days, 3, 6 and 12 months post-injury/illness. Demographic, clinical, radiological, laboratory, including hormonal data were collected.

**Results:**

A total of 91 adults, 56 (15 women/41 men) with TBI and 35 (27 women/8 men) with SAH were included. Perturbations in pituitary function were frequent early after the event but declined during the first year of follow-up. The most frequent deficiency was hypogonadotrope hypogonadism which was seen in approximately 25 % of the patients. Most of the variations were transient and without clinical significance. At 12 months, two patients were on replacement with hydrocortisone, four men on testosterone and one man on replacement with growth hormone. No relations were seen between hormonal levels and injury variables.

**Conclusions:**

Perturbations in pituitary function continue to occur during the first year after TBI and SAH, but only a few patients need replacement therapy. Our study could not identify a marker of increased risk of pituitary dysfunction that could guide routine screening. However, data demonstrate the need for systematic follow-up of pituitary function after moderate or severe TBI or SAH.

## Introduction

Traumatic brain injury and subarachnoid haemorrhage may cause permanent physical, cognitive, behavioural and psychosocial disabilities limiting daily activities [1–4]. With annual incidence rates from 235 to 538 cases per 100,000 inhabitants [5, 6] for TBI and of SAH from 2 to 23 cases per 100,000 inhabitants [7, 8], these injuries correspond to huge human and economic costs and offer significant challenges both with regard to acute care and follow-up.

There is accumulating evidence that specialised neurorehabilitation may enhance recovery and long-term outcome after acquired brain injury [9–12] and also that this should start in parallel with the acute care [13] in order to prevent secondary complications and enhance functional improvement.

Several medical complications, such as infection, hydrocephalus, respiration problems (tracheostomy), nutrition problems and heterotopic ossification may interfere with the post-acute rehabilitation process [14], while this remains unclear for endocrine dysfunction. Disturbed pituitary function has recently been highlighted as one potential factor that deserves attention and potentially treatment during neurorehabilitation [15–23]. However, until now, there is no consensus about either the prevalence, type or the clinical impact of pituitary disturbances after TBI and SAH and current recommendations for screening of pituitary function and replacement therapy are debated [24].

After TBI, some degree of post-traumatic hypopituitarism (PTHP) has been observed in 10–58 % of patients [16–18, 23, 25–29]. Several factors may affect the prevalence of PTHP including the time interval between TBI and evaluation of pituitary function, the severity of the injury and the diagnostic tests used [30]. In the chronic phase following TBI, growth hormone (GH), adrenocorticotrophic (ACTH), gonadotropin, and thyroidal deficiency as well as diabetes insipidus (DI) have been reported [16–18, 23, 25–28].

There are some corresponding studies of hormonal dysfunction after SAH and these reports on GH insufficiency, adrenal, gonadotropin, and thyroidal deficiency [19, 21, 22, 31]. The results of these are variable and even conflicting [19, 21, 22, 31–35] probably reflecting similar factors as discussed for TBI studies [30].

We have previously reported that pituitary deficiencies occur in a substantial number of patients early after TBI and SAH [36]. The aim of the current study was to evaluate the prevalence and course of pituitary function in patients during the first year after traumatic brain injury or subarachnoid haemorrhage and also to explore the relation between pituitary function and injury variables.

## Materials and methods

This report is based on prospectively collected data from a consecutive cohort of patients, admitted to the Neurointensive care unit (NICU) at Karolinska University Hospital from 1 March 2009 until 30 June 2012 after TBI or SAH. Patients were included at the NICU and followed at the Department of Rehabilitation Medicine at Danderyd University Hospital, Stockholm Sweden, at 3, 6 and 12 months post-injury/illness. Hormone testing was performed at Department of Endocrinology, Karolinska University Hospital. Inclusion was not performed during holidays for logistic and administrative reasons. Inclusion required a moderate or severe TBI or SAH due to a ruptured aneurysm, a lowest GCS score during the first day after the event of 3–13, age ≥18 years, living in the Stockholm region and obtained informed consent. For patients, who were unconscious or otherwise unable to give informed consent, the closest relative was asked.

The study was approved by Regional Ethical Review Board in Stockholm (No: 2008/3:9 2008/1574-31/3).

### Data collection

Demographic data (age, sex and smoking status), clinical and radiological parameters were prospectively collected from medical records at the NICU, Department of Rehabilitation Medicine and Department of Endocrinology, and transferred to the study data base.

#### Severity grading

In addition to the GCS score [37] (3–8 severe injury, 9–13 moderate injury), clinical severity in patients with SAH severity was graded according to the Hunt–Hess scale (HH) [38]: 1, asymptomatic or minimal headache, nuchal rigidity; 2, moderate to severe headache, no neurological deficit except for cranial nerve palsy; 3, drowsiness, confusion, mild focal deficit; 4, stupor, moderate to severe hemiparesis, early decerebrate; 5, deep coma, decerebrate posturing, moribund appearance. Aneurysms were verified by computed tomography angiography (CTA) or digital subtraction angiography (DSA). Aneurysms were divided into aneurysm from the anterior cerebral circulation and the posterior cerebral circulation.

Computed tomography (CT) lesion [39] grading of TBI was according to the CRASH model [40]: presence of one or more petechial haemorrhages (cerebral contusions), obliteration of the third ventricle or basal cisterns, subarachnoid bleed, midline shift >5 mm, and non-evacuated haematoma (subdural/epidural). Brain oedema, basilar skull fractures and facial fracture were added to the CT model.

CT grading of SAH was according to Fisher’s scale (1: no blood detected; 2: SAH less than 1 mm thick; 3: SAH more than 1 mm thick; and 4: intraventricular or parenchymal blood with or without diffuse SAH [39].

#### Assessment of endocrine function

An ACTH stimulation test (Synacthen test) and analyses of thyroid function, free thyroxine (fT4), thyroid-stimulating hormone (TSH) and free triiodothyronine (fT3), were performed 10 days post-injury or at discharge from NICU, if earlier. The lowest and highest serum osmolality and P-sodium values during this time period at NICU were also recorded.

**Fig. 1:**
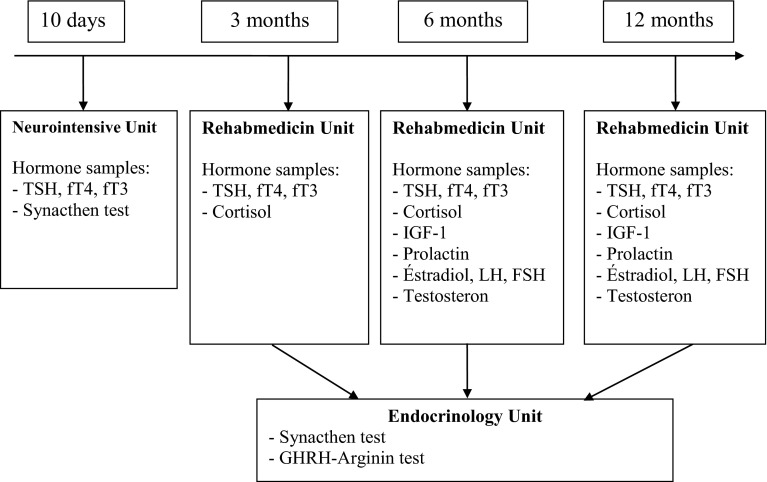
Flowchart of blood samples

After discharge from NICU blood was sampled at 3, 6 and 12 months, between 8 and 10 am. Flowchart of blood samples is presented in Fig. 1. At 3, 6 and 12 months blood was analysed for thyroid function (fT4, TSH and fT3) and cortisol. At 6 and 12 months, additional analyses were performed for insulin-like growth factor-1 (IGF-1), prolactin, estradiol in females, follicle-stimulating hormone (FSH) in females, luteinising hormone (LH) in females, and testosterone in males.

The optimal time for measurement of prolactin, IGF-1 and gonadal function after TBI and SAH is widely discussed. The time points 6 and 12 month were used in order to allow stabilisation of the pituitary function and thus also to allow relevant decisions on treatment.

At 3, 6 and 12 months post-TBI/SAH, P-sodium (P-Na) was measured and serum osmolality if P-Na level was below or above the normal reference range.

The protocol was designed according to our clinical experience and local resources. Thus, Synacthen test was performed in the Neurointensive care unit at 10 days when the patients were still in hospital. In contrast, measurement of basal cortisol was taken at 3, 6 and 12 months follow-up when patients were seen in the Rehabilitation outpatient clinic for clinical evaluation. The limit set for the Synacthen test at a morning cortisol <400 nmol/L was based on clinical experience combined with results from the literature [41–43].

ACTH stimulation test was performed by administration of Synacthen (250 µg) intravenously (i.v.), and blood samples were drawn before and 30 min after injection for analysis of cortisol. A normal response to the Synacthen test was defined as cortisol at 30 min >550 nmol/L. The patients were arbitrarily divided in three subgroups according to the cortisol response to the Synacthen test; one group with a subnormal response, one group with a cortisol response between 550 and 1000 nmol/L and one group with a cortisol response >1000 nmol/L.

To be able to manage the large amount of data and combinations of TSH, fT4 and fT3, hypothyroidism was defined as fT4 level below the normal reference range. Reference value for fT4 is presented in Table 1.

**Table 1:** Reference values for basal concentrations

	Reference values
TSH	0.4–3.5 mU/L (DxI)
fT4	8–14 pmol/L (DxI)
fT3	3.5–5.4 nmol/L (DxI)
Cortisol	≥400 nmol/L normal function
Cortisol (Synacthen test)	>550 nmol/L normal function
Estradiol	<600 nmol/L for follicular phase women
	200–2000 nmol/L for mid-cycle phase women
	300–1000 nmol/L for luteal phase women
	<150 pmol/L for post-menopausal women
FSH	2.5–10.0 U/L for follicular phase women
	4.0–14.0 U/L for mid-cycle phase women
	0.7–8.5 U/L for luteal phase women
	0.7–8.5 U/L for post-menopausal women
LH	1.8–12 U/L for follicular phase women
	18–90 U/L for mid-cycle phase women
	0.6–15 U/L for luteal phase women
	18–78 U/L for post-menopausal women
Testosterone	10–30 nmol/L for men
Prolactin	3–27 µg/L for age <50 years women
	3–20 µg/L for age >50 years women
	3–13 µg/L for men
IGF-1	250–610 μg/L for ages 18–19 years men
	210–600 μg/L for ages 18–19 years women
	250–590 µg/L for age 19–20 men
	220–550 µg/L for age 19–20 women
	160–420 µg/L years 20–25 years
	150–390 µg/L for ages 25–30 years
	140–370 µg/L for ages 30–35 years
	130–340 µg/L for ages 35–40 years
	120–320 µg/L for ages 40–45 years
	110–300 µg/L for ages 45–50 years
	110–270 µg/L for ages 50–55 years
	100–260 µg/L for ages 55–60 years
	90–240 µg/L for ages 60–65 years
	85–220 µg/L for ages >65 years
P-Sodium	137–145 mmol/L
Serum osmolality	280–300 mosmol/kg

*P* plasma, *U* unit

An age-dependent reference range (geometrical mean ± 2 SD) for IGF-1, independent of gender, was calculated based on the equation for the regression line in all patients: ^10^log [IGF-1 (µg/L)] = 2.581–0.00693 × age (year), with SD = 0.120 [44].

A growth hormone-releasing hormone—arginine stimulation test (GHRH-ARG) was performed in patients with IGF-1 <−2SD. GHRH-ARG stimulation test was performed by administration of GHRH 1-29 (1 mg/kg up to a maximum of 100 mg) i.v. at 0 min and then the infusion of arginine hydrochloride (0.5 g/kg up to a maximum of 30 g) i.v. from 0 to +30 min and blood samples for GH evaluation were taken every 15 min from −15 to +120 min. GH deficiency was presumed if GH—max was below 11.5 mg/L [Body Mass Index (BMI) <25], 8.5 mg/L (BMI 25–30), and 4.2 mg/L (BMI > 30) [45].

Gonadotropin dysfunction in post-menopausal women was defined as FSH, and LH or estradiol below the normal reference range, in pre-menopausal women in combination with amenorrhoea or oligomenorrhoea. The reference range for estradiol, FSH and LH is presented in Table 1.

Gonadotropin dysfunction in men was defined as testosterone below the normal reference range. The reference range of testosterone is presented in Table 1.

The reference range of prolactin, P-Na, serum osmolality, TSH and fT3 is presented in Table 1.

Analyses of cortisol, fT4, TSH, fT3, IGF-1, estradiol, FSH, LH, testosterone, GH, P-Na and serum osmolality were performed in the Department of Clinical Chemistry in Karolinska University Hospital using routine commercial kits.

#### Pituitary insufficiency during the first year after TBI or SAH

Patients were divided in groups of those, who showed hormonal disturbance at first or last tests, and those who did not show such disturbance. First test were taken at 10 days (cortisol and thyroid hormones) or 6 months (IGF-1, gonadotropin and prolactin). Last tests were taken at 12 months for all hormones.

We explore the relation between pituitary function and injury variables such the GCS score, pupil size or light reaction, peak of S100B (12–36 h), and hospitalisation length on the Neurointensive care unit for both TBI and SAH patients, and Fisher grade, Hunter and Hess grade for SAH patients, and CT scan pathology for TBI patients.

### Statistics

Statistical analysis was performed using IBM SPSS Statistics version 22 (IBM Corporation, Armonk, New York, USA).

This observational study reported frequencies as mean ± SD [range (min–max)] or number (per cent) or median (percentile) or median (min–max).

Nonparametric methods were used as data were not normally distributed according to Shapiro–Wilk’s test of normality. The Mann–Whitney *U* test was used for group comparisons of GCS, Hunt and Hess grade, Fisher grade and peak S100B (12–36 h). The Fisher’s exact test was used for comparisons of CT scan findings, pupil size at admission, pupil reaction to light stimulation at admission. The Wilcoxon Signed-Ranks test was used for comparison of hormone level. In all cases, significance level was set at *p* < 0.05.

The Mann–Whitney *U* test was used for non-response analysis with respect to these variables: age, BMI, GCS, Hunter and Hess scale, Fisher scale and peak S100B (12–36 h). The Fisher’s exact test was used for non-response analysis with respect to gender.

## Results

### Study population

In total 127 patients, 82 with TBI and 45 with SAH were included in the study.

**Fig. 2:**
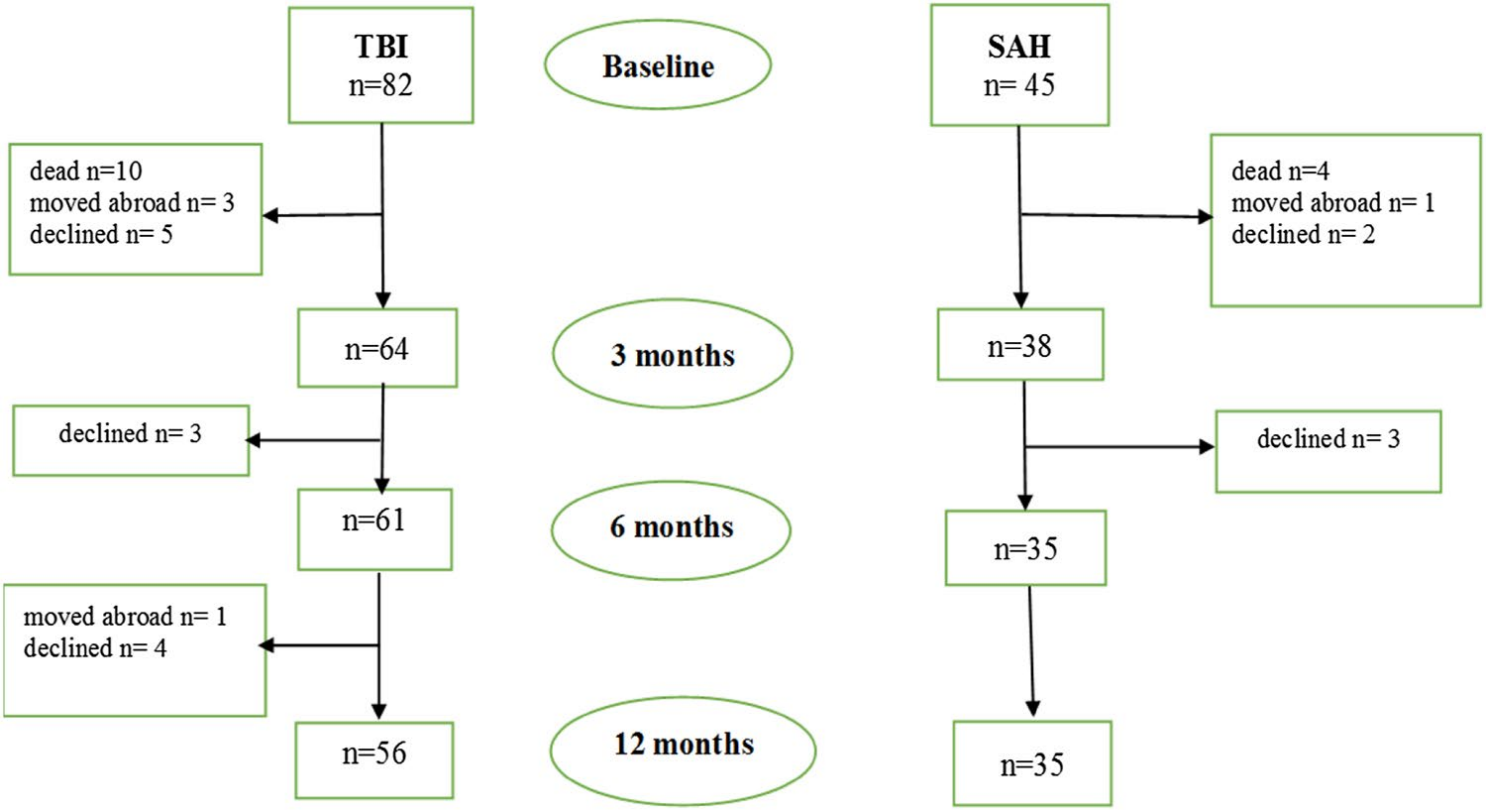
Flowchart of study participants

Ten patients with TBI and four with SAH died before 3 months follow-up. Twelve patients with TBI and five with SAH declined further participation in the study. Four patients with TBI and one with SAH moved abroad. A flowchart and drop-out of patients with TBI and SAH are presented in Fig. 2.

### Demographic and clinical characteristics

Of the 56 patients with TBI, 15 were women and 41 men, mean age was 47.1 ± 16.6 years, 44 patients had a severe brain injury and 12 patients a moderate brain injury according to the admission GCS score (mean GCS score 6.3 ± 2.9).

Of the 35 patients with SAH, 27 were women and 8 men, mean age was 57.4 ± 9.9 years, 22 patients had severe and 13 patients a moderate brain injury according to the admission GCS score (mean GCS score 7.9 ± 4.2). Most frequent Hunt and Hess scores at admission were 4 and 3. All patients had SAH visible on CT scan (Fisher grade 2–4). Data are displayed in Table 2.

**Table 2:** Baseline data of patients with traumatic brain injury and subarachnoid haemorrhage

	TBI *n* = 56	SAH *n* = 35
	(mean ± S.D.) (min–max)	(mean ± S.D.) (min–max)
Age, years	47.1 ± 16.6 (19–79)	57.4 ± 9.9 (28–76)
BMI	25.6 ± 4.8 (18.5–42.2)	25.7 ± 4.3 (20.3–36.5)
GCS	6.3 ± 2.9 (3–13)	7.9 ± 4.2 (3–13)
	*n* (%)	*n* (%)
Gender		
Male	41 (73.2 %)	8 (22.9 %)
Female	15 (26.8 %)	27 (77.1 %)
Smokers	19 (34.5 %)	16 (45.7 %)
GCS		
Moderate (9–13)	12 (21.4 %)	13 (37.1 %)
Severe (3–8)	44 (78.6 %)	22 (62.9 %)
Hunter and Hess grade		
1		1 (2.9 %)
2		3 (8.6 %)
3		14 (40.0 %)
4		15 (42.9 %)
5		2 (5.7 %)
Fisher grade		
1		0
2		3 (8.6 %)
3		8 (22.9 %)
4		24 (68.6 %)
Trauma cause		
Traffic accident	25 (44.6 %)	
Fall	28 (50 %)	
Assault	2 (3.6 %)	
Other	1 (1.8 %)	
CT scan		
Cerebral contusions	45 (80.8 %)	
Obliteration of the third ventricle or basal cisterns	15 (26.8 %)	
Subarachnoid bleed	45 (80.4 %)	
Midline shift >5 mm	21 (37.5 %)	
Subdural/epidural haematoma	48 (85.7 %)	
Brain oedema	12 (21.4 %)	
Basilar skull fractures	26 (46.4 %)	
Facial fracture	17 (30.4 %)	
Aneurysm localisation		
Anterior cerebral circulation AACA		
Anterior communicating artery ACoA		15 (42.9 %)
Middle cerebral artery MCA		8 (22.9 %)
Anterior choroidal artery AChA		1 (2.9 %)
Internal carotid artery ICA		3 (8.6 %)
Pericallosal artery		1 (2.9 %)
Posterior cerebral circulation APCC		
Posterior communicating artery PCoA		3 (8.6 %)
Basilar artery		1 (2.9 %)
Vertebral artery		1 (2.9 %)
Posterior inferior cerebellar artery PICA		1 (2.9 %)
AACA + APCC		
MCA + PCoA		1 (2.9 %)

*BMI* Body Mass Index (kg/m^2^)

### Endocrine evaluations

#### Hypothalamus–pituitary–adrenal (HPA) axis

In the acute phase 22/54 (41 %) TBI and 9/35 (26 %) SAH patients had low levels of morning s-cortisol. Results from the ACTH test were available for 53 patients with TBI and 34 patients with SAH. Six (11 %) patients with TBI and six (17 %) with SAH responded with subnormal increase in cortisol. Of these, five patients with TBI and all five patients with SAH had been treated with different types of steroids (methylprednisolone was used in the treatment of high intracranial pressure, and hydrocortisone for pharyngeal swelling and betamethasone was used in a few patients with SAH in the treatment of brain oedema).

In 18/53 patients (34 %) with TBI and 19/34 patients (56 %) with SAH the cortisol response was very high ranging from 1000 to 2000 nmol/L.

At 3 months follow-up 17/54 (32 %) TBI and 10/35 (29 %) SAH patients had low levels of morning cortisol. Results from the ACTH test were available for 10 patients with TBI and for 7 patients with SAH. Three patients with TBI and none with SAH responded with subnormal increase in cortisol. None of the patients with TBI and SAH had an exaggerated cortisol response over 1000 nmol/L. Ten patients refused to do the test or did not show up at the appointment.

At 6 months follow-up 19/52 (37 %) TBI and 23/34 (68 %) SAH patients had low levels of morning cortisol. Results from the ACTH test were available for 16 patients with TBI and for 19 patients with SAH. Only one patient with TBI and one patient with SAH responded with subnormal increase in cortisol. None of the patients with TBI and one with SAH had an exaggerated cortisol response. Seven patients refused to do the test or did not show up at the appointment.

At 12 months follow-up 18/52 (35 %) TBI and 15/35 (43 %) SAH patients had low level of morning cortisol. Results from the ACTH test were available for 11 patients with TBI and for 11 patients with SAH. Two patients with TBI and none with SAH responded with subnormal increase in cortisol. None of the patients with TBI and two patients with SAH had an exaggerated cortisol response. Eleven patients refused to do the test or did not show up at the appointment.

Replacement therapy was clinically indicated in two patients with TBI and cortisol insufficiency after 12 months follow-up.

**Fig. 3:**
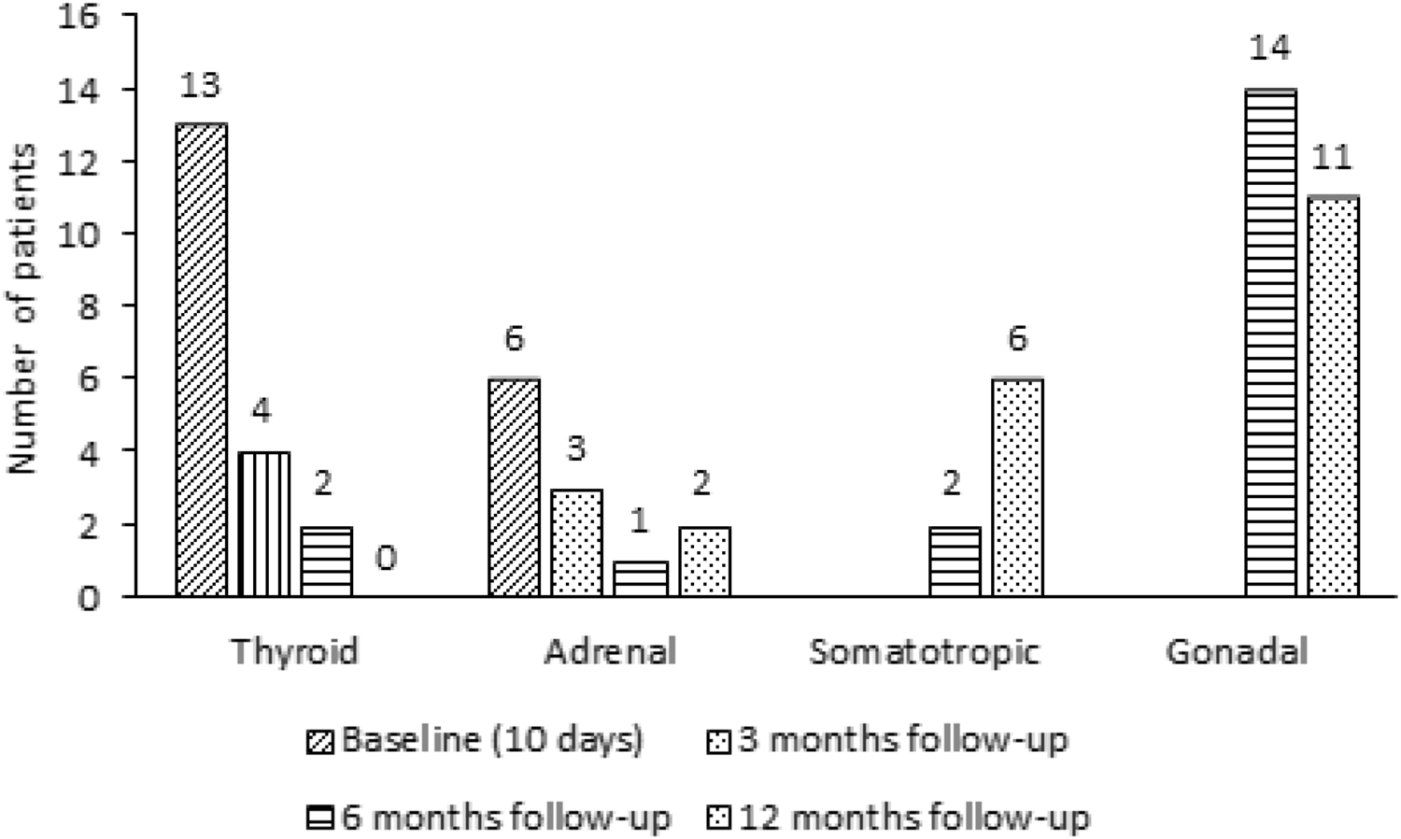
Numbers of patients with pituitary deficiency after TBI at baseline and 3, 6 and 12 months follow-up

**Fig. 4:**
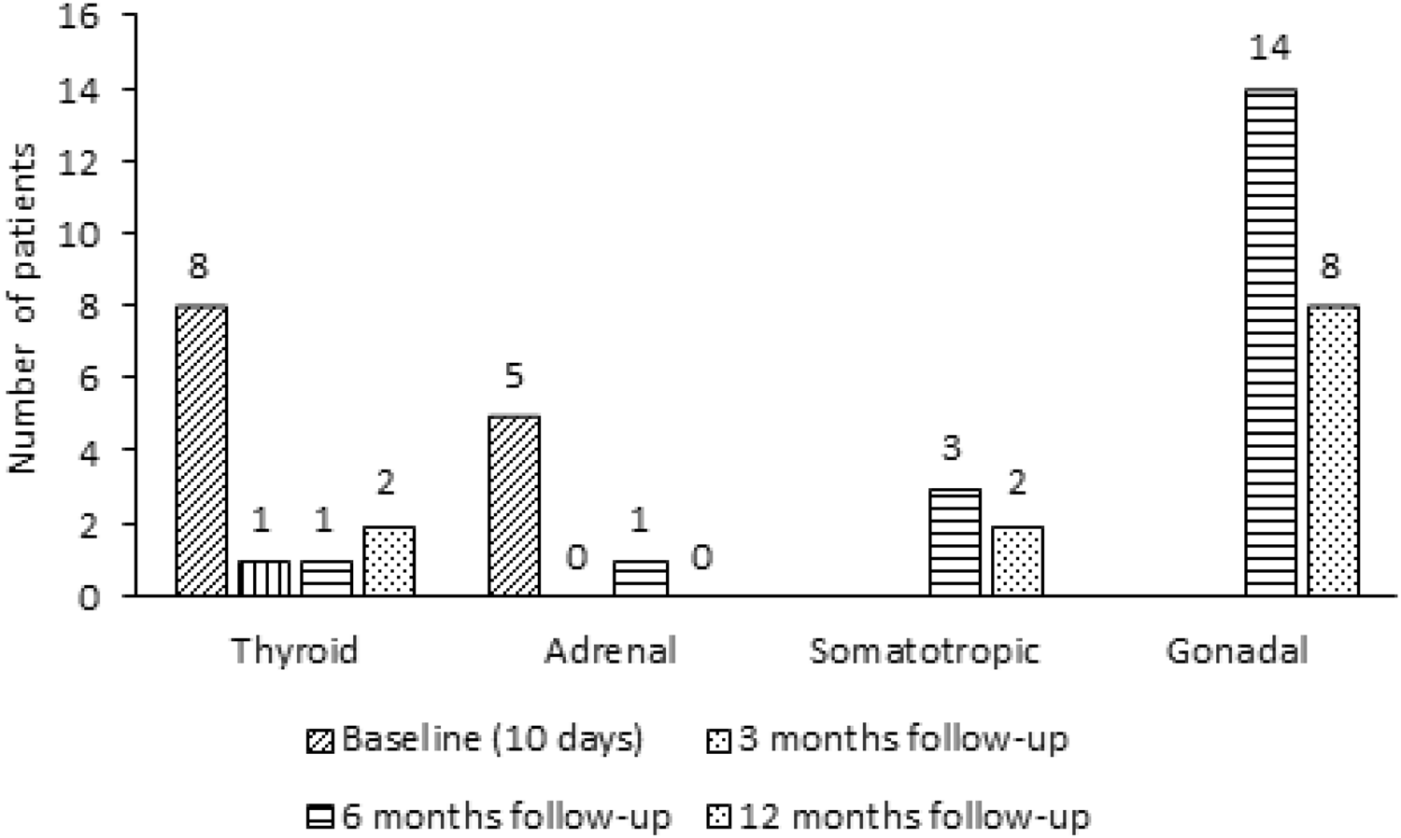
Numbers of patients with pituitary deficiency after SAH at baseline and 3, 6 and 12 months follow-up

Numbers of patients with insufficiency of the HPA axis after TBI and SAH at baseline and 3, 6 and 12 months follow-up are displayed in Figs. 3 and 4.

#### Thyroid function

In the acute phase, biochemical hypothyroidism was seen in 13/55 (23.6 %) patients with TBI and in 8/35 (22.9 %) patients with SAH. Ten TBI patients with low fT4 had normal TSH, one had low TSH and one increased TSH. Four SAH patients with low fT4 had normal TSH, three had low TSH and one increased TSH.

At 3 months follow-up 4/54 (7.1 %) TBI and 1/35 (2.9 %) SAH patients had low levels of fT4. All TBI patients with low fT4 had normal TSH, but one SAH patient had increased TSH.

At 6 months follow-up 2/52 (3.8 %) TBI and 1/34 (2.9 %) SAH patients had low levels of fT4. All TBI and one SAH patients with low fT4 had normal TSH.

At 12 months follow-up 0/52 (0 %) TBI and 2/35 (5.7 %) SAH had low levels of fT4.

All SAH patients with low fT4 had normal TSH.

Replacement therapy was not clinically indicated in any patient.

Numbers of patients with biochemical hypothyroidism after TBI and SAH at baseline and 3, 6 and 12 months follow-up are displayed in Figs. 3 and 4.

#### Somatotropic function

At 6 months follow-up low levels of IGF-1 (<−2SD) were observed in 2/52 (4 %) of patients with TBI and in 3/34 (9 %) patients with SAH.

At 12 months follow-up low levels of IGF-1 (<−2SD) were observed in 6/52 (12 %) patients with TBI and in 2/35 (6 %) patients with SAH.

Dynamic assessment at 12 months follow-up using GHRH-ARG stimulation test in 4/6 TBI and 0/2 SAH patients showed that one patient with TBI had growth hormone deficiency (GHD).

Only this patient received replacement with growth hormone after 12 months follow-up.

Number of patients with low IGF-I levels (somatotropic dysfunction) after TBI and SAH at baseline and 3, 6 and 12 months follow-up are displayed in Figs. 3 and 4.

We also observed a high level of IGF-1 (>+2SD) in 9/52 (17 %) patients with TBI and in 4/34 (12 %) patients with SAH at six months and in 7/52 (14 %) patients with TBI and in 1/35 (3 %) patient with SAH at 12 months.

#### Gonadotropin function

At 6 months follow-up, low testosterone levels were observed in 12/37 (32 %) patients with TBI and in 3/8 (38 %) patients with SAH. At 12 months follow-up low testosterone levels were observed in 9/39 (23 %) patients with TBI and in 3/8 (38 %) patients with SAH.

Replacement therapy with testosterone was clinically indicated in two patients with TBI and two patients with SAH after 12 months follow-up.

At 6 months follow-up low FSH and low LH or low estradiol levels were observed in 2/15 (13 %) women with TBI and in 11/26 (42 %) women with SAH. At 12 months follow-up low FSH and low LH levels were observed in 2/13 (15 %) women TBI and in 5/27 (19 %) women with SAH.

In post-menopausal women with gonadotropins insufficiency oestrogen therapy was not initiated.

Number of patients with gonadal dysfunction after TBI and SAH at baseline and 3, 6 and 12 months follow-up are displayed in Figs. 3 and 4.

#### Prolactin

At 6 months follow-up, low prolactin levels were observed in 1/52 (2 %) patient with TBI and in 1/34 (3 %) patient with SAH. At 12 months follow-up, low prolactin levels were observed in 2/51 (4 %) patients with TBI and in 0/35 (0 %) patients with SAH.

At 6 months follow-up, high prolactin levels were observed in 3/51 (6 %) patients with TBI and in 0/34 (0 %) patients with SAH. At 12 months follow-up high prolactin levels were observed in 4/51 (8 %) patients with TBI and in 4/35 (11 %) patients with SAH.

#### Antidiuretic hormone

In the acute phase 63/84 (75 %) of patients with TBI and 40/46 (90 %) of patients with SAH had P-Na > 146 and serum osmolality >300 mosm/kg. Of these 9 TBI and 9 SAH patients had been treated with fludrocortisone, and 7 patients with TBI and 10 with SAH had been treated with desmopressin.

In 10/82 (12 %) patients with TBI and in 2/46 (4 %) patients with SAH a low P-Na in combination with a low serum osmolality was observed. Both patients with SAH lost in weight (−6.1 to −1.4 kg) while six patients with TBI lost weight (−7.3 to −1.1 kg) and four increased in weight (+1.5 to +7.1 kg).

Transient perturbations in P-Na, serum osmolality and body weight were seen in a few patients during follow-up but the number of patients was too few to draw any statistical conclusions. Only short-term interventions were required in these patients.

### Relations between pituitary insufficiency during the first year after TBI or SAH and acute injury characteristics

Seven/46 patients (15.2 %) with TBI and four/30 patients (13.3 %) with SAH had an insufficient response to ACTH stimulation during the first year after TBI or SAH. Data from 10 TBI and 5 SAH patients were missing.

There were no significantly differences between patients with or without cortisol a sufficient response with regard to injury variables for both TBI and SAH patients. Data are displayed in Table 3.

**Table 3:** Relations between cortisol dysfunction during the first year after TBI or SAH and acute injury variables

	TBI		SAH	
	Cortisol dysfunction	Normal cortisol function	Cortisol dysfunction	Normal cortisol function
	*n* = 7	*n* = 22	*n* = 4	*n* = 7
	Median (q25–q75)	Median (q25–q75)	Median (q25–q75)	Median (q25–q75)
GCS	7.0 (7.0–8.0)	5.5 (3.0–10.0)	8.0 (3.0–13.0)	8.0 (5.0–13.0)
Hunter and Hess			3.0 (3.0–3.75)	3.0 (2.0–4.0)
Fisher scale			4.0 (4.0–4.0)	4.0 (3.0–4.0)
Peak of S100B (12–36 h)	0.43 (0.22–0.64)	0.30 (0.19–0.50)	0.37^a^	0.23 (0.13–0.24)
Length of stay in NICU	33.0 (20.0–35.0)	20.0 (9.0–27.5)	23.5 (16.8–31.0)	19.0 (13.0–26.0)

*q25–q75* quartiles 25 and 75, respectively

^a^ *n* = 2

Twelve/51 patients (23.5 %) with TBI and nine/35 patients (25.7 %) with SAH had low levels of fT4 during the first year after TBI or SAH. Data from 5 TBI patients was missing.

Length of stay in NICU was longer for the TBI patients with low fT4 levels (*p* = 0.004) than in TBI patients with a normal thyroid function, but not for SAH patients.

There were no significantly differences between patients with or without low fT4 levels with regard to others injury variables for both TBI and SAH patients. Data are displayed in Table 4.

**Table 4:** Relations between thyroidal dysfunction during the first year after TBI or SAH and acute injury variables

	TBI		SAH	
	Thyroidal dysfunction	Normal thyroid function	Thyroidal dysfunction	Normal thyroid function
	*n* = 12	*n* = 33	*n* = 9	*n* = 20
	Median (q25–q75)	Median (q25–q75)	Median (q25–q75)	Median (q25–q75)
GCS	7.0 (4.5–10.25)	5.0 (3.5–8.0)	5.0 (3.0–7.0)	8.0 (4.25–13.0)
Hunter and Hess			4.0 (3.0–4.0)	3.5 (3.0–4.0)
Fisher scale			4.0 (3.5–4.0)	4.0 (3.0–4.0)
Peak of S100B (12–36 h)	0.36 (0.20–0.51)	0.30 (0.17–0.49)	0.24 (0.11–0.31)	0.09 (0.06–0.22)
Length of stay in NICU	27.0 (20.3–35.8)**	18.0 (8.5–21.0)	20.0 (16.0–35.0)	16.0 (12.25–24.0)

** *p* < 0.01

Six/48 patients (12.5 %) with TBI and four/34 patients (11.8 %) with SAH had low IGF-1 during the first year after TBI or SAH. Data from 8 TBI and 1 SAH patients were missing.

A higher peak of S100B (12–36 h) was noticed in TBI patients with low IGF-1 (*p* = 0.003) than in TBI patients with a normal IGF-1, but not for SAH patients.

There were no significantly differences between patients with or without low IGF-1 with regard to others injury variables for both TBI and SAH patients. Data are displayed in Table 5.

**Table 5:** Relations between somatotropic dysfunction during the first year after TBI or SAH and acute injury variables

	TBI		SAH	
	Somatotropic dysfunction	Normal somatotropic function	Somatotropic dysfunction	Normal somatotropic function
	*n* = 6	*n* = 33	*n* = 6	*n* = 25
	Median (q25–q75)	Median (q25–q75)	Median (q25–q75)	Median (q25–q75)
GCS	6.5 (4.75–7.25)	7.0 (4.0–9.0)	5.5 (3.0–11.75)	6.0 (3.5–13.0)
Hunter and Hess			3.0 (1.25–4.0)	3.0 (3.0–4.0)
Fisher scale			3.0 (2.0–4.0)	4.0 (3.5–4.0)
Peak of S100B (12–36 h)	0.61 (0.53–1.22)**	0.28 (0.17–0.47)	0.10 (0.04–0.22)	0.21 (0.08–0.33)
Length of stay in NICU	17.0 (11.0–29.0)	16.0 (6.5–28.0)	14.0 (11.5–15.0)	19.0 (15.0–27.0)

** *p* < 0.01

Sixteen/48 (28.6 %) with TBI (3 women, 13 men) and 14/34 (40 %) with SAH (10 women, 4 man) developed gonadotropin insufficiency during the first year after TBI or SAH. Data from 8 TBI and 1 SAH were missing.

The GCS score was lower in SAH patients with gonadotropin insufficiency (*p* = 0.022) than in SAH patients with a normal gonadotropin, but not for TBI patients.

There were no significantly differences between patients with or without gonadotropin insufficiency with regard to others injury variables for both TBI and SAH patients. Data are displayed in Table 6.

**Table 6:** Relations between gonadotropin dysfunction during the first year after TBI or SAH and acute injury variables

	TBI		SAH	
	Gonadotropin dysfunction	Normal gonadotropin function	Gonadotropin dysfunction	Normal gonadotropin function
	*n* = 16	*n* = 32	*n* = 14	*n* = 18
	Median (q25–q75)	Median (q25–q75)	Median (q25–q75)	Median (q25–q75)
GCS	5.5 (3.0–9.5)	7.0 (4.25–8.75)	12.0 (5.0–13.0)*	5.0 (3.0–8.0)
Hunter and Hess			3.0 (3.0–4.0)	4.0 (3.0–4.0)
Fisher scale			4.0 (3.0–4.0)	4.0 (3.0–4.0)
Peak of S100B (12–36 h)	0.45 (0.15–0.56)	0.29 (0.18–0.53)	0.15 (0.07–0.28)	0.21 (0.09–0.29)
Length of stay in NICU	17.0 (9.5–24.5)	19.0 (9.0–26.5)	17.5 (14.5–30.0)	19.0 (14.5–23.0)

* *p* < 0.05

Two/47 patients (4.3 %) with TBI and one/34 patient (2.9 %) with SAH had low prolactin during the first year after TBI or SAH. Five/47 patients (10.6 %) with TBI and four/34 patients (11.8 %) with SAH had high prolactin during the year after TBI or SAH. Data from 10 TBI and 1 SAH patients were missing.

### Non-response analysis/bias

We found no significant difference in gender, age, BMI, GCS, Hunter and Hess and Fisher scale in participants with missing data for each hormone group.

## Discussion

*In the present study, we found that although perturbations in pituitary function are frequent early after the event, they tend to wear off even though some persist or even occur later during the first year after TBI and SAH.* The hormonal levels were very varying and low as well as high levels of all hormones were observed. However, in most cases, the changes in hormone levels were transient and without clinical relevance. Thus, *in total, 7 out of 95 patients required hormonal replacement* (4 testosterone, 1 growth hormone, 2 hydrocortisone) after 12 months while none needed treatment for hormonal hypersecretion. None of the patients had pan-hypopituitarism.

Overall, our results are mainly in accordance with the findings in some previous studies of TBI [17, 18, 23, 28]. E.g. Agha et al. [17] observed growth hormone deficiency in 13 % of 50 patients at 6 months and in 10 % at 12 months; corresponding figures for adrenal insufficiency were 18 % at 6 months and 18 % at 12 moths, respectively; for gonadotropin deficiency 23 % and 13 %, for thyroidal deficiency 2 % and 2 % at these time points, respectively. Tanriverdi et al. [18] observed hyposomatotropism, hypocortisolism, hypogonadism and hypothyroidism in 38, 19, 8 and 6 % of 52 patients at twelve months after TBI. Klose et al. [23] observed pituitary insufficiency in 76 % of 46 patients in the early phase post-injury, in 13 % at 3 months and 6 months post-injury, and in 11 % at 12 months. Bavisetty et al. [28] observed in 70 patients at 3 months and 6–9 months post-TBI major hormonal deficits in 25 % and 21 %, respectively, in the somatotroph, gonadotroph, thyrotroph, corticotroph axis.

Similarly, our findings are roughly in accordance with previous studies after SAH. Tanriverdi et al. [19]. observed GH deficiency in 36 % of 22 patients at 12 months after SAH and deficient adrenocorticotrophic hormone (ACTH) in 14 %. Furthermore, in 84 patients, Khajeh et al. [22] found gonadotropin deficiency in 20 and 5 % and GH deficiency in 10 and 5 % at 6 and 14 months, respectively, after SAH. A recent study by Kronvall et al. [21] of 60 patients at 6–12 and at 12–24 months after SAH. At these time points, they observed ACTH deficiency in 20 and 11 %, GH dysfunction in 20 and 29 %, and gonadotropin dysfunction in 2 and 11 %, respectively.

Thus, our study as well as previous studies demonstrates that pituitary perturbations are most common early after the injury but may occur in a substantial proportion of patients at later stages. Obviously, prevalence figures differ between studies and this probably reflects differences in study samples as well as with regard to definitions of pituitary hypofunction and analyse methods [23].

*The most frequent pituitary insufficiency at the 1* *year follow*-*up, observed in approximately one fourth of the patients in our study, was hypogonadotropic hypogonadism.* Generally, the frequency of insufficient hormonal levels declined during follow-up and replacement was only clinically relevant in a few patients. This is in contrast to the findings in some earlier studies, reporting higher frequencies, which could be due to differences in defining pituitary hypofunction, differences in the analyses of hormones and differences in study samples.

In our study, we also observed periodic increases in hormone levels as well as high stimulated levels of cortisol after ACTH injection. The high responses to ACTH stimulation could indicate a persistent stress in these patients as also indicated by the high prolactin level seen in some patients. Of notice, high responses in the TBI group was only seen during the first three months after the event, while high responses in the SAH group was also seen 6 and 12 months after the event indicating a more persisting stress in patients with SAH.

During follow-up, the frequency of patients with perturbations in hormonal levels in our study was similar for patients with TBI or SAH, indicating that the affection of the pituitary function was not dependent on aetiology. These conditions may share some pathophysiological mechanisms such as vascular support of the pituitary [46–48].

Although this study is subject to limitations, we believe it adds some valuable information to the existing literature and that one strength of our study is the comprehensive and long-term data collection. Consistent data now demonstrate that affection of pituitary function is most frequent in the early phase after the TBI and SAH and declines during the following months [5–7, 10, 11]. Only a small proportion of patients with moderate or severe TBI or SAH need replacement therapy but these patients must be diagnosed and treated.

Unfortunately, we did not find an injury marker that could help identify patients at risk and thus to support a routine for selective screening of pituitary function. We investigated only a few injury markers and believe this is an important area for further studies. Recently, Zheng et al. [49] suggested that pituitary apparent diffusion coefficient (ADC) is a sensitive and independent marker of pituitary damage following TBI and others have started to investigate genetic markers [50].

However, taken together with previous reports, we find it justified to suggest that all patients with moderate or severe TBI or SAH should be routinely screened at least at one time point after the injury and that screening around 1 year after the injury may be considered a minimal obligation. However, screening of the HPA axis, TSH-thyroid axis and FSH/LH-sex hormones every third month until 12 months and of GH-IGF-1 at 12 months after the event may well be considered. Further, testing when there is clinical suspicion should be liberal. Obviously more studies are needed in order to develop evidence-based guidelines in this area. The potential impact of pituitary perturbations on the long-term outcome after TBI and SAH also need further attention.

### Study limitations

Regarding drop-outs and missing data, there is a risk of underestimation of pituitary disturbances in our study samples. However, most participants had some further contact with the study team as well as with other caregivers, and clinically significant insufficiency might then have been suspected and diagnosed.

The GHRH-ARG stimulation test was only performed in patients with S-IGF-1 < −2SD. This might potentially increase the risk of underestimating growth hormone deficiency which might in addition be underestimated in patients with hypothalamic damage.

## Conclusions

Perturbations in pituitary function continue to occur during the first year after TBI and SAH, and low as well as high hormonal levels are seen. The changes in hormone levels are often transient and without clinical relevance. Thus, 1 year after TBI in total 7 out of 95 patients needed hormonal replacement. However, it is important to realise that a persistent hypofunction of the pituitary requiring hormonal replacement might occur in a minority after TBI or SAH. This has to be considered in the clinical management of these patients although there is still a need for more data to allow evidence-based guidelines.
